# The midwives service scheme: a qualitative comparison of contextual determinants of the performance of two states in central Nigeria

**DOI:** 10.1186/s41256-016-0017-4

**Published:** 2016-10-24

**Authors:** Arnold I. Okpani, Seye Abimbola

**Affiliations:** 1 London School of Hygiene and Tropical Medicine, London, United Kingdom; 2 National Primary Health Care Development Agency, Plot 681/682, Port-Harcourt Crescent, Area 11, Garki, Abuja, FCT Nigeria; 3 University of Sydney, School of Public Health, Rm 324, Edward Ford Building A27, Sydney, NSW 2006 Australia

**Keywords:** Human resources for health, Decentralisation, Community, Primary health care, Nigeria

## Abstract

**Background:**

The federal government of Nigeria started the Midwives Service Scheme in 2009 to address the scarcity of skilled health workers in rural communities by temporarily redistributing midwives from urban to rural communities. The scheme was designed as a collaboration among federal, state and local governments. Six years on, this study examines the contextual factors that account for the differences in performance of the scheme in Benue and Kogi, two contiguous states in central Nigeria.

**Methods:**

We obtained qualitative data through 14 in-depth interviews and 2 focus group discussions: 14 government officials at the federal, state and local government levels were interviewed to explore their perceptions on the design, implementation and sustainability of the Midwives Service Scheme. In addition, mothers in rural communities participated in 2 focus group discussions (one in each state) to elicit their views on Midwives Service Scheme services. The qualitative data were analysed for themes.

**Results:**

The inability of the federal government to substantially influence the health care agenda of sub-national governments was a significant impediment to the achievement of the objectives of the Midwives Service Scheme. Participants identified differences in government prioritisation of primary health care between Benue and Kogi as relevant to maternal and child health outcomes in those states: Kogi was far more supportive of the Midwives Service Scheme and primary health care more broadly. High user fees in Benue was a significant barrier to the uptake of available maternal and child health services.

**Conclusion:**

Differential levels of political support and prioritisation, alongside financial barriers, contribute substantially to the uptake of maternal and child health services. For collaborative health sector strategies to gain sufficient traction, where federating units determine their health care priorities, they must be accompanied by strong and enforceable commitment by sub-national governments.

## Background

Nigeria is a lower middle income country with a population of about 180 million people [1]. Maternal and child health outcomes in Nigeria are among the worst in the world [2, 3]. In 2013, maternal mortality was estimated at 576/100,000 live births and under-five mortality was estimated at 128/1000 live births [4]. There are however marked urban–rural and inter-regional differences: outcomes are worse in rural areas and in Northern Nigeria compared to urban areas and Southern Nigeria [4, 5]. In 2008, under-five mortality in rural areas was estimated at 191/1000 live births, compared to 121/1000 live births in urban areas [6]. The estimate for North-East zone of Nigeria was 222/1000 live births, compared to 89/1000 in the South-West zone [6]. Notably, Nigeria did not meet any of the health-related millennium development goals (MDGs), except for the reversal of the spread of HIV/AIDS [3, 7].

Contributing to the poor state of maternal and child health in Nigeria, especially in rural areas, is the widespread shortage of trained health workers [8, 9]. Between 2008 and 2013, 47 % of women in rural areas received antenatal care from a skilled provider, compared to 86 % of women in urban areas [4]. In the same period, only 23 % of births in rural areas were attended by a skilled birth attendant, compared to 67 % in urban areas [4]. To address the human resource gaps and accelerate progress toward the MDGs, the federal government of Nigeria introduced the Midwives Service Scheme (MSS) in 2009 [10]. Implemented through the National PHC Development Agency (the federal agency responsible for policy guidance to sub-national governments for the implementation of PHC nationally), the MSS was intended to fill the health care human resource gaps and rapidly increase the availability of skilled birth attendants especially in rural communities. It was also intended to improve the uptake of antenatal care, postnatal care, routine immunization and other primary health care (PHC) services [10].

Because Nigeria is a federation in which the federating units (i.e. state governments) can determine their health care priorities [11, 12], MSS was designed as a collaboration between the three tiers of government: federal, state and local governments. Each tier of government had roles and responsibilities outlined in a memorandum of understanding (MOU), signed by all parties in each jurisdiction. Midwives are recruited into the scheme by the federal government, and are then trained and deployed by the federal government to pre-determined PHC facilities in rural communities. Recruited midwives were retired or unemployed midwives, as well as new graduates from schools of midwifery around Nigeria [13]. Retired midwives who were still able to work potentially had an additional incentive to participate in the scheme as they were paid for providing services within the MSS in addition to any ongoing pension payments. To ensure that 24-h obstetrics services were available in MSS PHC facilities, the midwives were deployed in groups of four to each facility. Community health extension workers (CHEWs) were also recruited and deployed in pairs to each MSS facility to support the midwives and engage in community mobilization [14]. Communities that had MSS facilities were supported by the government to form community health committees to act as a bridge between the facility and the community. Four MSS facilities were situated in each local government area (LGA) that was selected to participate in the scheme. These four facilities were linked to a secondary care facility (general hospital) to handle referrals from the MSS facilities; a hub-and-spoke design [13].

In creating the scheme, the federal government of Nigeria was responding to local and international pressure to reign in its embarrassing maternal and child health situation and make appreciable progress toward the MDGs [13]. What was perhaps overlooked in the build up to the scheme was the central role that state governments play in the political economy of Nigeria [11]. Nigeria’s federal constitution grants substantial powers to federating units – states – to determine their priorities in matters such as health care. Although states (and the local governments within them) do receive federal funds, they are not accountable to the federal government in determining their health care spending choices and the federal government is limited to the use of advocacy to secure states and local government support for health sector strategies agreed or conceptualised at the federal level [11]. See Table 1 for an illustration of how health sector responsibilities are shared among the tiers of government in Nigeria.

**Table 1:** A simplified illustration of the sharing of health sector responsibilities by the tiers of government in Nigeria

Responsibility		Tier of Government			Comment
		FG	SG	LG	
Health policy making		***	**	—	Whilst the FG leads, SG participate through the National Council on Health
Regulation	Price	***	**	—	FG determines salary scales. SG can decide to adopt it or not. User fees are determined separately by FG and SG
	Quality	***	*	—	FG sets health workers training curricula, licenses practitioners, facilities and commodities. SG participates in enforcement
	Quantity	**	*	—	FG and SG control location of public sector facilities. There is generally very little control over number of practitioners trained
Resource generation		***	**	*	LG lacks capacity to invest substantially in human capital development and health infrastructure
Planning, budgeting and resource allocation		***	**	*	A substantial share of the FG health budget is spent in providing support to SG and LG
Service provision	Primary Care	*	**	***	Primary care is provided at all levels but most of the primary health care responsibilities lie with the LG
	Secondary Care	**	***	—	Secondary care provision also happens at tertiary level health facilities
	Tertiary Care	***	**	—	Many SG own tertiary level facilities, typically affiliated to universities as teaching hospitals
Monitoring and evaluation		***	**	**	All tiers have established M&E mechanisms

*FG* federal government, *SG* state government, *LG* local government, *** mostly responsible, ** partly responsible, * minimally responsible, − not responsible. For the purpose of simplicity, the roles played by private sector and donor organisations are excluded from the table

Source: Okpani AI; Abimbola S. 2015 [44]

In implementing the MSS, the MOU detailing the agreement of the federal government with sub-national governments included sharing the salary payment of the midwives among federal, state and local governments in a ratio of 3:2:1, and for local governments to provide accommodation for the midwives [13]. The midwives were employed for an initial one-year period, but the MSS was designed so that subject to satisfactory performance, their appointments would be renewed annually. In addition, the MSS was designed with the intent that when the project expires, state governments would take over from the federal government and implement the scheme in partnership with local governments.

The allocation of MSS facilities and midwives to states was based on the relative burden of maternal mortality in the states. States in the North-East and North-West that had the highest mortality burdens received proportionately more midwives than states in the south of Nigeria [14]. In total, about 1000 of the over 28,000 PHC facilities [15] throughout Nigeria were designated as MSS facilities. These 1000 facilities were clustered around 250 general hospitals. Previous reports indicate that there have been improvements in the use of maternal and child health services in communities where MSS facilities are located [13, 14, 16]. However, these improvements have not significantly changed maternal and child health outcomes nationally due to the limited coverage of the scheme, and the continued preference for informal home deliveries in areas that contribute the most to maternal deaths [13, 17]. The MSS is also currently faced with challenges ranging from declining funding support from all levels of government, insecurity, high attrition of midwives, and scarcity of midwives in certain regions. Federal funding for the scheme declined from 3.6 billion naira (US$22.7 million) in 2012 to 1.5 billion naira (US$9.7 million) in 2013 [18, 19]; a 58 % funding shortfall in one calendar year. The effect of declining federal funding on the scheme varies between states, based on their willingness to sustain the scheme and other local factors [20].

Previous studies have examined the determinants of the availability and retention of MSS midwives in the communities where they were posted, including irregular and insufficient salary payments, temporary job status, poor accommodation and working conditions, and being posted far from their family [13, 20]. But none has specifically explored contextual factors in the states, LGAs and host communities that impact on the uptake of the scheme and that might explain differences in uptake between states. In this study, we take a qualitative approach to examine differences in uptake of the MSS (in terms of political support and community response) in Benue and Kogi – two neighbouring and largely similar states in central Nigeria [21, 22]. The study aimed to use the explanation of how local context influence the availability, utilisation and sustainability of the MSS, to develop recommendations that could guide policy makers in improving existing and future maternal and child health interventions in Nigeria and other low- and middle-income countries with a similar federal structure of government.

## Methods

This comparative qualitative study was conducted between 18 June and 14 July 2015. In-depth interviews (IDIs) were used to explore the reasons for differences in maternal and child health prioritization in the face of obvious need. Focus group discussions (FGDs) were used to examine factors that influence choice of place of delivery in communities that have health facilities staffed by skilled birth attendants, and explore the perceptions of mothers on the value of services provided by the health workers. The analysis was conducted using thematic content analysis [22].

### Sampling strategy

Benue and Kogi are neighbouring states in central Nigeria with similar socio-economic characteristics, but which differ markedly in their maternal and child health outcomes, with Kogi showing better outcomes in nearly all indicators (Table 2) [4, 21]. Within the two states, LGAs were selected based on the presence of MSS clusters; i.e. four designated MSS PHC facilities linked to a secondary care hospital. Communities in which FGDs were held were selected based on the presence of an MSS PHC facility. For the IDIs, senior government health officials who are or were involved in the design and implementation of the MSS were selected from LGA and state government health departments in Benue and Kogi, as well as from the federal government. At the federal level, respondents for IDIs were selected purposively [23] based on prior information of the federal coordination of MSS. Selection of interviewees at state and LGA level was also purposive to include ‘information-rich’ participants [24], with an element of snowball sampling [22] as interviewees recommended their colleagues who had a good grasp of the implementation of MSS. Efforts were made to ensure that government officials interviewed in Benue and Kogi were of similar number and position. On the other hand, women who had babies in communities with an MSS facility were selected as participants in the FGDs, recruited based on the criteria that they must be resident in a community with an MSS PHC facility, be a mother that had a baby (facility or home delivery) within the past year at the time of the interview, and be able to communicate in English or the Nigerian Pidgin English. Recruited by the most senior health worker at the MSS facility who are usually well known and trusted by the community, the FGD participants were purposively selected to include women who had had both facility and non-facility deliveries to allow for a diversity of perspectives [25] – see Table 3 for details of FGD participants.

**Table 2:** Selected socioeconomic, maternal and child health indicators for Benue and Kogi

Domain	Indicator	Benue	Kogi
Education	Proportion of Males with no education – Male (%)	11.1	14.1
	Proportion of Females with no education – Female (%)	25.7	25.7
	Literacy rate – Male (%)	92.6	91
	Literacy rate – Female (%)	52.8	71.6
Employment	Proportion currently employed – Male (%)	76.8	71.8
	Proportion currently employed – Female (%)	77.5	71.3
Maternal Health	Antenatal care coverage (%)	57.4	87.5
	Received 2 or more doses of tetanus toxoid in last pregnancy (%)	40.3	79
	Place of delivery – Health facility (%)	50.9	78.9
	Place of delivery – Home (%)	48.3	18.8
	Delivery attended by skilled birth attendant (%)	51.6	70.9
	Had postnatal check up in the first 2 days after birth (%)	39.4	71.2
Child Health	Vaccination coverage (%): children, 12–23 months, with all basic vaccinations	20	35.6

Source: Data from the Nigeria Demographic and Health Survey 2013 [4]

**Table 3:** Summary of focus group discussion participants

Location	Age	Level of education	Number of children	Place of delivery of the last child	Who attended the last delivery
Benue	25	Junior secondary 3	1	Hospital	Community Health Extension Worker
	20	Senior secondary	1	Home	Traditional Birth Attendant
	24	Senior secondary	1	PHC facility	Midwife
	24	None	2	PHC facility	CHEW
	42	None	9	PHC facility	CHEW
	20	Senior secondary	2	Hospital	Midwife
	23	Senior secondary	1	PHC facility	Midwife
	36	Senior secondary	2	Hospital	Doctor (Caesarean section)
	21	Primary	2	PHC facility	CHEW
	27	Post-secondary	2	PHC facility	CHEW
Kogi	18	Senior secondary	1	PHC facility	Midwife
	26	Senior secondary	2	PHC facility	Midwife
	19	Senior secondary	1	PHC facility	Midwife
	21	Senior secondary	2	Home	Traditional Birth Attendant
	20	Senior secondary	1	PHC facility	Midwife
	27	Post-secondary	2	Home	Traditional Birth Attendant
	21	Senior secondary	2	PHC facility	Midwife
	28	Post-secondary	2	Home	Traditional Birth Attendant
	38	Senior secondary	4	PHC facility	Midwife
	26	Senior secondary	2	PHC facility	Midwife

### Data collection

To ensure a degree of uniformity, the same interview guide was used for all IDIs, but the questions were tailored to the level of government at which the interviewee worked. Participants were, however, allowed to lead the discussion with the researcher probing as necessary to enhance the richness and depth of the data provided [25]. The interviews explored perceptions of participants on the design, implementation and financing of the MSS and the roles of the different tiers of government in the process. All IDIs were conducted in English and took place in the respondents’ offices. Interviews lasted between 30 min and an hour. The FGD explored the reasons for the participants’ choice of health provider and their views on the services of the midwives. The same discussion guide was used in both states for the FGDs. Because both states have people of different ethnic and language groups, the FGDs were conducted in English or Nigerian Pidgin English, which is closest to a lingua franca in the region and in much of Nigeria [25, 26]. Each FGD had 10 participants. Due to time constraints and the logistical difficulties of finding a suitable location in rural Nigeria, the FGDs were conducted in the MSS facilities. Each FGD lasted about one hour. In all, twelve IDIs and two FGDs were conducted and were all audio recorded. The recordings were subsequently transcribed. However, immediately after the interviews and group discussions, notes were written to document perceptions and thoughts on body language, expressions and other nuances from the interaction. This self-debriefing process was also an opportunity to evaluate the discussion, and reflect on themes that emerged. The interview and discussion guides were refined based on emerging concepts while topics for exploration in subsequent sessions were noted. These notes provided context for the interpretation and understanding of the data produced.

### Data analysis

An inductive thematic approach was adopted for this research [27] as the objectives centred on eliciting from the participants their views on the uptake on the MSS in their respective states, LGAs, and communities. Thematic analysis is sufficient to allow a comparison of views and perceptions on the ‘reason for the situation’ in Benue and Kogi. It is also useful to explore what women in communities with MSS facilities thought of the scheme and what informed their decisions to use (or not use) those services. Data analysis followed the steps outlined by Green and Thorogood [22]: ‘familiarization with the data, identifying codes and themes, coding the dataset, and organising codes and themes’. The first few transcripts were read and coded by AIO. The codes were then collated and arranged in categories and subcategories. Emerging themes were identified, and these themes were thereafter used to code the rest of the dataset. New themes that emerged were applied, and those deemed redundant or repetitive were subsequently dropped. A Microsoft Excel workbook was used to organise and sort the codes. Each sheet in the workbook were assigned a category of code, subcategories were delineated within each sheet. Sections of coded transcript were then cut and pasted in the relevant sheets of the workbook. Relevant sections of the field notes were added to each segment to create context. This method allowed for ease of comparison within and across categories and the visualization of the entire coded dataset. The categories and subcategories were examined for similarities and differences in views expressed. To ensure the reliability and credibility of the analysis, the data was triangulated by comparing and contrasting the response of participants from the same and from different study locations. While the initial analysis was conducted by AIO, the final codes and themes were reviewed by SA with reference to the transcripts.

### Ethics

Ethics approval for the study was provided by the National Health Research Ethics Committee of Nigeria, and the London School of Hygiene and Tropical Medicine. Participation in the study was entirely voluntary and based upon the participant signing a written informed consent form. In line with the terms of consent to which participants agreed, all participants have been de-identified, by removing information on name, community, and local government of participants. Participants agreed at the beginning of each FGD to maintain confidentiality within the group by not discussing outside the group individual opinions raised by others during discussions [27, 28].

## Results

The themes that emerged from the sessions are framed around the support for the scheme by sub-national governments and the uptake of the scheme by women in rural communities.

### Support for the scheme by sub-national governments

Based on the interviews, state government support for MSS was the most important determinant of the uptake of the scheme in the states. This includes state government support for timely and regular payment of state counterpart funds for the salary of the midwives, integration of state funding for the scheme into the states’ annual recurrent health care budget, regular monitoring and supervision to ensure quality and reduce absenteeism, and supply of commodities. Kogi had integrated funding for the scheme into its health budget and was identified as one of the states that have been most consistent in payment of allowances to the midwives from the outset of the programme. Health care in general was considered by respondents to be of high priority in Kogi. In the words of an interviewee in Kogi: *They* [*the state government*] *have been paying them* [*midwives on the MSS*] *from the beginning*… *The governor is a health friendly governor*, *he has been supporting the programme. The previous governor was also health friendly*. In contrast, Benue has not been meeting any of its obligations to the MSS. Interviewees highlighted that since inception, Benue has not paid its counterpart funding to finance the salary of the midwives. One federal interviewee said that in Benue “*the midwives are not paid any money* [*by the state*] *right from the beginning*… *They complain they don’t have money to support the MSS*, *but maybe it is the political support which was lacking*.”

While there was a marked contrast between support for the scheme at the state level in Benue and Kogi, the situation at the LGA level was different. Respondents reported similar levels of support at the LGA level in each state. Most LGAs in each state had paid allowances to the midwives until recently. However, some LGAs in Kogi paid higher allowances than recommended in the MOU. In both states, LGAs were working with communities to provide accommodation to midwives, although the accommodation provided was not always ideal. There was also a lack of understanding of the scheme by administrative departments of LGAs in both states as they sometimes withheld the payment of their part of the salary of the midwives on the grounds that the midwives were supposed to be in place for only one year. In Benue, an interviewee said *the LGA stopped their salary last year*, *[stating] that their tenure was over after one year… That generated a lot of problems… and we resolved the issue by getting the federal people to give them renewal letters.* The limited understanding of the scheme was linked to the limited involvement of sub-national governments in the design and implementation of MSS. There was a sense among state and LGA interviewees that much of the decisions are made at the federal level with limited input from sub-national governments. For example, sub-national governments in Benue and Kogi did not have the flexibility to reassign midwives to PHC facilities based on utilisation patterns. One participant in Benue said: “*We have particular facilities that are in dire need of midwives, but because they are posted directly from the federal government, we lack that capacity to reassign them*.”

However, in spite of their limited role, state and LGA interviewees viewed the scheme favourably and saw the MSS as a welcome boost to their maternal and child health efforts. The fact that was MSS implemented in all the states of Nigeria divided opinions, even though states differed in the number of clusters they had, based on their maternal and child mortality burdens. For example, one federal respondent questioned the assumption that the scheme would be perceived as relevant in all the states: “*I don’t think most states knew really what their problem was that made the project necessary… It was a top-down project, conceived at the federal level and pushed down to the states… without consideration of each state’s peculiar needs*.” On the contrary, other respondents emphasised the need for such a scheme that cuts across all states, given the common health care human resource shortfalls in rural communities across Nigeria. A federal interviewee said “*The shortage of midwives is nationwide. When we did the initial appraisal, no facility out of 652 PHC facilities reviewed [nationally], had four midwives… close to 80% of facilities did not even have a single midwife.*” Likewise, another federal interviewee emphasised the need for a national scheme by saying “*We had tertiary hospitals with consultants in obstetrics and paediatrics in their tens or dozens delivering about 300 babies a year*; *and we would have no doctor in a PHC facility delivering 200 to 300 babies in a month*.” The top-down nature of the MSS puts the federal government in a position to take the lead in implementing the MOU, while it lacks the constitutional power to enforce the terms of the MOU on sub-national governments. One federal interviewee said “*We tend to persuade them [states], we tend to encourage them, but there are no mechanisms for sanctioning. Where we had challenges of them not implementing the MOU, we were left with no other options*.”

The divisions in opinion about the necessity of the scheme in all states of Nigeria also had implications for its long-term sustainability. At the outset, the scheme was intended to be supported with funding from all three tiers of governments for an initial period of two years after which the federal government was to discontinue funding. States and LGAs were required to continue funding the scheme and formally integrating eligible midwives into the state public service. Respondents again had divergent views on the capacity and willingness of state governments to sustain the scheme on the long term. A federal interviewee said: “*The states can fund the scheme. That they don’t do it does not mean that they can’t. It all depends on fiscal discipline and prioritization*.” The view that states were not getting their health care priorities right was strongly supported by respondents across all three tiers. To illustrate this, a respondent in Benue said that the Benue state government had been building general hospitals across the state for years; and a federal interviewee said “*If states reprioritized… and stopped building big general hospitals or fancy diagnostic centres… without a commensurate expenditure on PHC, they would be able to afford the scheme very easily…*” There was little hope that Benue will prioritise PHC, especially with the existing restrictions on employment into the state civil service for about a decade, including of PHC workers. When asked which tier of government should sustain the scheme on the long term, one Benue state interviewee said: “*Federal of course. The state is not employing. It is the federal government that should employ and deploy them to the states. If they hand it over to the state, the thing will collapse.*” On the contrary, Kogi respondents were surer of the willingness of their state government to sustain the MSS. When asked if Kogi can sustain the MSS, an interviewee responded: “*Of course… they have the will to sustain it… the state should be able to continue the scheme.*”

In Kogi and Benue, LGA respondents wanted the federal government to take responsibility for continuing the scheme. They preferred the present arrangement where the federal government bore the bulk of the responsibility for hiring, training, deploying, and paying the midwives. The reason given was that the LGAs lacked the financial capacity to employ highly skilled health workers such as nurses, midwives and doctors because of their wages. Indeed, it was reported that the health department is usually the largest of LGA departments, taking up most of the personnel costs. Thus LGAs are only able to employ lower skilled health workers such as community health extension workers; an additional reason for state governments to take responsibility for the scheme. Respondents also reported that in the few instances in both Kogi and Benue that midwives from the MSS had somehow been able to enter the civil service, they were deployed away from the PHC facilities to secondary care facilities run by the state governments. In the words of one federal interviewee:

*If you go to LGAs and disaggregates the distribution of overhead costs, you will discover that out of all the departments in the LGA, the PHC department takes close to 60 % or more. So already the administrators at that level are saying; “look, health is taking so much and it is the same health that requires more hands”. So, it becomes more difficult. That’s why I say for this level, if the state can take that responsibility, it will be a huge step in the right direction*.

### Uptake of the scheme in the communities

Notwithstanding mixed perceptions about the need for the scheme and sub-optimal sub-national support, interview and discussion participants were convinced of the benefits of the MSS in Benue and Kogi. The benefits attributed to the scheme included improvement in the availability of health workers in rural communities, reduction in maternal and child deaths, increased antenatal care attendance and deliveries in PHC facilities. The midwives from the MSS constituted the vast majority of skilled birth attendants in the LGAs where they are deployed. And in some instances they were the only midwives available to take deliveries in entire LGAs. One interviewee in Benue said:

*They [midwives] are helping in some communities that we didn’t even have hope of any midwife ever going to work in… Before they came, when women went into labour, people will carry them with wheel barrow because some of those places have no good roads. Or they will carry them on bicycles to a health facility or general hospital far away. Before they will get there, the woman will die on the road, or the baby will die in the uterus. There had been such cases before but now with the help of MSS midwives it has been reduced*.

On their part, mothers who participated in the FGDs said they found the scheme very helpful in improving pregnancy outcomes and child health. They reported that having the midwives available round the clock was really helpful as they could find them in the health facility whenever they needed to go there. Some of the participants who delivered at night or early in the morning stated that they had no difficulty getting help when they arrived at the health facility. In Benue, one of them said: “*In the old days, women used to die when they are delivering a baby… With the presence of the midwives, that is largely avoided. Not that people don’t die, but it’s not as common as it used to be.*” Participants in the FGDs reported that there was marked increase in number of people using the health facilities following the arrival of the MSS midwives. Because of the superior skills of the midwives, most of the mothers preferred getting care for themselves and their family at the MSS PHC facilities, instead of private health facilities and patent medicine dealers operating in the communities. The following is part of a discussion during the FGD in Benue:

Woman 3: *I prefer to have a midwife conduct a delivery than home delivery because the midwives are trained in school specifically for that purpose. The ones at home are just doing things… I don’t really know how to put it. There are many side-effects; sometimes they don’t even wear gloves… they don’t even use it at all. They don’t even take care to protect the baby from contracting an illness, the mother too. They don’t know anything pertaining to that.*

Woman 7: *Their own is only to remove the baby for you*…

Woman 4: *Jack the baby out*!

Woman 5: *Sometimes if you deliver in the clinic, you will not be able to deliver well, they will assist you. If you are having difficulty, they can aid you [give episiotomy]. If it’s at home, you will not be able to deliver*.

Woman 6: *I prefer to come here because they are trained in school. The chemists [patent medicine dealers] learnt as apprentices. Some didn’t even learn; just because they can read the name of a medicine they start acting as chemists. But these ones went to school to acquire the knowledge. That is why I prefer government clinics like this one. They will take care of you and give you drugs*.

Woman 4: *I’ve been in this community long enough to know how things run. I prefer coming here because if you come here, when they try and they see that the illness is beyond their capacity, they will quickly refer you. The other private clinics here will be trying until you give up*.

Many of the women who had home deliveries felt that delivering in the health facility with a midwife attending was in the woman’s best interest. They had planned to deliver in the health facility but could not get there when they went into labour either because the “*baby came too quickly*” or there was no emergency transportation. However, one woman who delivered at home in the Benue group said she deliberately decided to deliver at home because she felt she was strong enough to do so. On what she had planned to do if things did not go according to plan, she said she had stocked-up on sugar with which to make a solution to give her more strength. In discussing the reasons why some people do not use the health facility, the Kogi FGD highlighted that the distance of some of the settlements to the health facility was a significant barrier. The problem is compounded by the poor state of the roads and lack of emergency transportation. Notably, money was not regarded as a significant barrier by the Kogi group. They reported that they had to pay between 500 and 1000 naira (US$ 2.5–5) for an uncomplicated delivery in the MSS facility if they had purchased delivery items beforehand. This was in agreement with the fee reported by one of the state health officials interviewed. In contrast, the most frequently cited barrier at the Benue FGD was cost of treatment, and distance was mentioned as a less important barrier. Participants reported that the fee for antenatal care and having an uncomplicated delivery ranged from 4000 to 5000 naira (US$ 20–25). Many women opt for home delivery because they could not afford the fees charged at the health facility. But one participant in the group commented that sometimes people do not go the health facility out of ignorance not necessarily because of poverty.

## Discussion

In exploring the basis for differential uptake of maternal and child health services within the MSS in Benue and Kogi in central Nigeria, this study identified two major themes: prioritisation of PHC by sub-national governments and financial barriers to care at the community level. The differences between Benue and Kogi in complying with the terms of the MOU agreed by the three tiers of government at the beginning of the MSS is such that Kogi pays its counterpart funds for the scheme while Benue does not. The results also suggest a difference in broader priorities, beyond the MSS, in health care financing in both states. These differences are situated in the context of the roles the three tiers of government in Nigeria have in the sharing of health care responsibilities as shown in Table 1. There is a tendency for states to invest more in ‘visible’ health infrastructure such as construction of hospitals while neglecting PHC which caters to the health care needs of majority of Nigerians, especially the rural poor. This pro-rich inequity in health care resource allocation is in line with previous studies in Nigeria and elsewhere [9, 29, 30].

The findings of this study suggest that the success and long term sustainability of the MSS or any nationally coordinated PHC intervention depends heavily on the support it receives from state governments. Nigerian states do not always align (or have to align) with federal health care strategies and priorities. The successes noted in Kogi relative to Benue were attributed to the supportive disposition of the current state governor and his predecessor. Nigerian state governors can seriously impair or facilitate the achievement of national health goals. For example, the drive to eradicate polio was slowed for several years partly due to the decision, in 2003, by then governor of Kano, the most populous state in Nigeria, to stop the administration of the polio vaccine in Kano [31]. Efforts by the federal government to persuade the governor failed, and it took the intervention of UNICEF, WHO and Islamic leaders from around the world to convince the Kano governor of the safety of the polio vaccines.

While the situation in Benue is not as dramatic as the Kano scenario, the lack of support for maternal and child health at the PHC level has real consequences for the people of Benue as shown in Table 2. Beyond the MSS, Benue also lags behind Kogi and most other states in other PHC strengthening initiatives. Benue is yet to establish a state PHC board, a strategy for strengthening PHC by bringing PHC governance, implementation, monitoring and evaluation under one state-level authority thereby reducing the sort of fragmentation and inefficiency demonstrated in this and previous studies on the governance of human resources for PHC in Nigeria [32, 33]. States with state-level PHC governance have seen rising budget allocation to PHC and increased use of maternal and child health services [34].

The mothers in communities hosting MSS facilities in both states were, unsurprisingly, very supportive of the scheme. Whilst both groups generally agreed that user fees can deter some women from using health services, they differed markedly on their views on how much of a barrier that can constitute. Mothers in Benue felt that delivery fees were such a big deterrent that many women are opting to deliver at home, but the Kogi group felt it was not much of a problem. The potential for high user fees to disproportionately exclude the poor from using health services is well documented in past studies [35, 36] and might explain the differences in views expressed by the groups. With delivery fees in Benue five to ten times higher than in Kogi, the willingness or ability to pay may be a bigger issue in Benue than it is in Kogi. This finding should be interpreted with caution as it represents the opinion of a handful of non-randomly selected mothers: to what extent, therefore, this finding is generalizable to the larger populations in both states is beyond the scope of this study and is well worth exploring in further studies.

Interviews conducted at the federal level were elite interviews [37] and respondents were often pressed for time. The state and LGA interviewees may have responded to the study as a ‘federal evaluation of the management of MSS at the state or LGA level’ given that the federal PHC agency endorsed the study. These circumstances may have biased the responses to the interviews. In addition, due to time and logistic constraints, participants in the group discussion were recruited by health workers and the discussions were held in the health facility premises. This may have also biased the content of the discussions. Further, conducting the group discussions in Pidgin English restricted the discussions to semi-literate and literate women, a scenario that may have shaped the views held and expressed at the discussion sessions. Given time and resource constraints, this study cannot claim to have covered the full breadth of contextual factors that account for differential support for the MSS and uptake of maternal and child health services in Benue and Kogi. That was not the objective of this study. Rather, it has generated plausible explanations that can be taken into consideration in designing and implementing interventions which involve collaboration between tiers of government in Nigeria especially for the purpose of addressing inequities. This, however, does not imply generalisability of the findings beyond the context of this study.

Given the restricted role the federal government plays in setting the agenda for health care in states, there is a need for more creative ways of forging useful partnership with state governments. The opportunity for more sustainable, and enforceable collaboration with state governments is presented by the 2014 National Health Act [38] which gives the federal government more power (and more funds through a Basic Health Fund) to intervene in PHC [39]. The Basic Health Fund can only be accessed by states that meet set criteria, and this provision can be used to persuade the states to refocus on maternal and child health. In addition, the Act empowers the federal government to directly address health care human resource challenges in Nigeria [40].

With the vast majority of Nigerians without access to any form of prepaid health care and instead paying out of pocket [41], any intervention with the objective of significantly scaling up the use of maternal and child health services must include mechanisms for keeping out-of-pocket payment as low as is practicable, including universal health coverage with financial protection for the poor [42, 43]. The MSS provides a model for achieving this, but state governments have to go beyond the attraction of the more visible physical infrastructure of hospitals to strengthening institutions to deliver PHC services to the majority of their populations.

## Conclusion

In a constitutional federal democracy, where the federating units determine their health care priorities, subnational governments can substantially enhance or undermine the outcome of a national health care human resource strategy with serious consequences for the target populations. Nigeria’s Midwives Service Scheme represents a good model for improving availability of highly skilled health workers in previously underserved communities. Its impact has, however, been markedly limited by suboptimal commitment by some participating subnational governments as demonstrated in this study by contrasting outcomes in Benue and Kogi states; two Nigerian states with similar socioeconomic characteristics. Differential levels of political support and prioritisation, alongside financial barriers occasioned by relatively high user fees, contributed substantially to affect the uptake of maternal and child health services. Consequently, for strategies aimed at improving PHC and addressing inequities to be successful in this setting, it must be accompanied by a total and unwavering commitment by subnational governments. It should, to the same degree, be able to address demand side constraints especially those relating to user fees. While the MSS was designed as strategy to achieve the health-related MDGs, insights on why Nigeria failed to achieve the goals should inform efforts to achieve the new Sustainable Development Goals. Notably, it is important to prioritise engagement with sub-national stakeholders, and to include sub-national indicators of progress towards the new goals as a way to potentially encourage competition among sub-national jurisdictions.

## Abbreviations

FGD: Focus group discussion
IDI: In-depth interviews
MDGs: Millennium development goals
MOU: Memorandum of understanding
MSS: Midwives service scheme
PHC: Primary health care

## References

[1] The World Bank. Nigeria [Internet]. 2015. Available from: http://data.worldbank.org/country/nigeria. Accessed 1 July 2016.

[2] Hogan MC, Foreman KJ, Naghavi M, Ahn SY, Wang M, Makela SM (2010). Maternal mortality for 181 countries, 1980–2008: a systematic analysis of progress towards Millennium Development Goal 5. Lancet.

[3] World Health Organization (2015). World health statistics 2015.

[4] National Population Commission (NPC) [Nigeria] and ICF International, International NPC and ICF. Nigeria Demographic and Health Survey 2013. Abuja, Nigeria, and Rockville, Maryland, USA; 2014.

[5] Barros AJ, Ronsmans C, Axelson H, Loaiza E, Bertoldi AD, Franca G V, et al. Equity in maternal, newborn, and child health interventions in Countdown to 2015: a retrospective review of survey data from 54 countries. Lancet. 2012/04/03 ed. 2012;379(9822):1225–33. 10.1016/S0140-6736(12)60113-5 22464386

[6] National Population Commission (NPC) [Nigeria], ICF Macro, Macro NPC and ICF (2009). Nigeria Demographic and Health Survey 2008.

[7] National Bureau of Statistics Nigeria, NBS (2015). Millennium Development Goals Performance Tracking Survey Report 2015.

[8] Bangdiwala SI, Fonn S, Okoye O, Tollman S (2010). Workforce Resources for Health in Developing Countries. Public Health Rev.

[9] Abimbola S, Olanipekun T, Igbokwe U, Negin J, Jan S, Martiniuk A (2015). How decentralisation influences the retention of primary health care workers in rural Nigeria. Glob Heal Action.

[10] National Primary Health Care Development Agency Nigeria, NPHCDA (2010). The MDG-DRG Funded Midwives Service Scheme (MSS) Baseline Report.

[11] Asoka T (2015). Political leadership change is not a system change: Overcoming the inertia of “change” the Nigerian health sector. Africa Heal Niger.

[12] Suberu R, Suberu R. The Nigerian federal system: performance, problems and prospects. J Contemp African Stud. 2010;28(4):459–77.

[13] Abimbola S, Okoli U, Olubajo O, Abdullahi MJ, Pate MA. The midwives service scheme in Nigeria. PLoS Med. 2012;9:e1001211. 10.1371/journal.pmed.1001211 PMC3341343 22563303

[14] National Primary Health Care Development Agency Nigeria (2012). The MDG-DRG Funded Midwives Srevice Scheme: Concept, Process and Progress.

[15] Federal Ministry of Health Nigeria, Health FM of (2013). A Directory of Health Facilities in Nigeria 2011.

[16] Partnership for Reinforcing Routine Immunisation in Northern Nigeria-Maternal/Newborn and Child Health (PRRINN-MNCH). The midwives service scheme [Internet]. Health Service Delivery. PRRINN-MNCH; 2014. Available from: http://www.prrinn-mnch.org/documents/PRRINN-MNCH2MidwivesSchemeSummary.pdf. Accessed 1 July 2016.

[17] Ononokpono DN, Odimegwu CO. Determinants of Maternal Health Care Utilization in Nigeria: a multilevel approach. Pan Afr Med J. 2014;17 Suppl 1:2. 10.11694/pamj.supp.2014.17.1.3596 PMC3958146 24643545

[18] NPHCDA (2014). Annual Programme Implementation Report 2013.

[19] XE. XE Currency Charts (USD/NGN) [Internet]. 2015. Available from: http://www.xe.com/currencycharts/?from=USD&to=NGN&view=5Y. Accessed 1 July 2016.

[20] Olujimi S, Onovo A, Marsden P, Okechukwu E, Burlew R, Jaskiewicz W (2014). Study of Attrition, Availability, and Retention of Midwives Service Scheme Officers in Nigeria.

[21] Online Nigeria. Kogi State [Internet]. Available from: http://www.onlinenigeria.com/links/kogiadv.asp?blurb=305. Accessed 1 July 2016.

[22] Green J, Thorogood N (2014). Qualitative Methods for Health Research. Third.

[23] Pope C, Mays N (1995). Reaching the parts other methods cannot reach: an introduction to qualitative methods in health and health services research. BMJ.

[24] Patton MQ (1990). Designing Qualitative Studies.

[25] Guest G, Namey EE, Mitchell ML (2013). Focus groups.

[26] Goglia F. Nigerian Pidgin English [Internet]. Manchester Working Group on Language Contact. The University of Manchester; 2010. Available from: http://languagecontact.humanities.manchester.ac.uk/McrLC/casestudies/FG_NigerianPidgin.html

[27] Wilkinson S, Silverman D (2004). Focus group research. Qualitative Research, Theory, Methods and Practice.

[28] Green J, Thorogood N (2014). Responsibilities, ethics and values.

[29] Burstein R, Wollum A, Dwyer-Lindgren L, Gakidou E. Primary health care in Nigeria: a systematic subnational analysis of levels and trends in maternal and child interventions 2000–13. Lancet Glob. 2015;3(Supplem):S21. 10.1186/s12916-015-0438-9 PMC4557921 26329607

[30] Rohde J, Cousens S, Chopra M, Tangcharoensathien V, Black R, Bhutta ZA (2008). 30 years after Alma-Ata: has primary health care worked in countries?. Lancet.

[31] Jegede AS. What led to the Nigerian boycott of the polio vaccination campaign? PLoS Med. 2007;4(3):e73. 2007/03/29 ed. 10.1371/journal.pmed.0040073 PMC1831725 17388657

[32] Wonodi C, Onoja R, Erchick D, Meghani A, Ng C, Umeh C (2014). Narrative Report of Primary Health Care Under One Roof (PHCUOR) Scorecard.

[33] Abimbola S, Negin J, Jan S, Martiniuk A (2014). Towards people-centred health systems: a multi-level framework for analysing primary health care governance in low- and middle-income countries. Heal Policy Plan.

[34] Sokpo E, McKenzie A. Primary Health Care Under One Roof; Reflections from our experience in Jigawa State Nothern Nigeria [Internet]. Kano, Nigeria: Partnership for Reviving Routine Immunization in Nothern Nigeria; Maternal, Newborn and Child Health Initiative (PRRINN-MNCH); Available from: http://www.prrinn-mnch.org/documents/PHCUOR.pdf. Accessed 1 July 2016.

[35] Jacobs B, Ir P, Bigdeli M, Annear PL, Van Damme W (2012). Addressing access barriers to health services: an analytical framework for selecting appropriate interventions in low-income Asian countries. Heal Policy Plan.

[36] Babalola S, Fatusi A (2009). Determinants of use of maternal health services in Nigeria--looking beyond individual and household factors. BMC Pregnancy Childbirth.

[37] Green J, Thorogood N (2014). In-depth interviews.

[38] Nigeria National Assembly. National Health Act [Internet]. 2014. Abuja: National Assembly of the Federal Republic of Nigeria; 2014. Available from: http://www.herfon.org.ng/Official%20Gazette%20of%20the%20National%20Health%20Act.compressed.pdf

[39] Uzochukwu B, Onwujekwe O, Mbachu C. Implementing the Basic Health Care Provision Fund in Nigeria, A framework for accountability and good governance [Internet]. Resilient and Responsive Health Systems (RESYS). Online: Health Policy Research Group; 2015. Available from: http://resyst.lshtm.ac.uk/sites/resyst.lshtm.ac.uk/files/docs/reseources/Nigeria-brief.pdf

[40] Okpani AI. Confronting weak health workforce governance in Nigeria. Africa Heal Niger. 2015;37(4):7–8. Available from: http://africa-health.com/wp-content/uploads/2015/05/Nigeria-edition-May-2015.pdf.

[41] McIntyre D, Ranson MK, Aulakh BK, Honda A. Promoting universal financial protection: evidence from seven low- and middle-income countries on factors facilitating or hindering progress. Heal Res Policy Syst. 2013;11(36):36. 10.1186/1478-4505-11-36 PMC3848816 24228762

[42] Onoka CA, Onwujekwe OE, Uzochukwu BS, Ezumah NN. Promoting universal financial protection: constraints and enabling factors in scaling-up coverage with social health insurance in Nigeria. Heal Res Policy Syst. 2013;11:20. 10.1186/1478-4505-11-20 PMC3686590 23764306

[43] World Health Organization (2010). World Health Report: health systems financing: the path to universal coverage [Internet].

[44] Okpani AI, Abimbola S. Operationalizing universal health coverage in Nigeria through social health insurance. Niger Med J. 2015;56(5):305–10. 10.4103/0300-1652.170382 PMC4698843 26778879

